# Gut microbiome resilience of green-lipped mussels, *Perna canaliculus,* to starvation

**DOI:** 10.1007/s10123-023-00397-3

**Published:** 2023-07-31

**Authors:** Siming Li, Tim Young, Stephen Archer, Kevin Lee, Andrea C. Alfaro

**Affiliations:** 1 Aquaculture Biotechnology Research Group, School of Science, Faculty of Health and Environmental Sciences, Auckland University of Technology, Private Bag 92006, Auckland, 1142 New Zealand; 2 The Centre for Biomedical and Chemical Sciences, School of Science, Faculty of Health and Environmental Sciences, Auckland University of Technology, Private Bag 92006, Auckland, 1142 New Zealand; 3 Department of Environmental Science, School of Science, Faculty of Health and Environmental Sciences, Auckland University of Technology, Private Bag 92006, Auckland, 1142 New Zealand

**Keywords:** Green-lipped mussels, Starvation, Microbiome, Marine microbiome, Microbial ecology, 16S rRNA

## Abstract

Host gut microbiomes play an important role in animal health and resilience to conditions, such as malnutrition and starvation. These host-microbiome relationships are poorly understood in the marine mussel *Perna canaliculus*, which experiences significant variations in food quantity and quality in coastal areas. Prolonged starvation may be a contributory factor towards incidences of mass mortalities in farmed mussel populations, resulting in highly variable production costs and unreliable market supplies. Here, we examine the gut microbiota of *P*. *canaliculus* in response to starvation and subsequent re-feeding using high-throughput amplicon sequencing of the 16S rRNA gene. Mussels showed no change in bacterial species richness when subjected to a 14-day starvation, followed by re-feeding/recovery. However, beta bacteria diversity revealed significant shifts (PERMANOVA *p*-value < 0.001) in community structure in the starvation group and no differences in the subsequent recovery group (compared to the control group) once they were re-fed, highlighting their recovery capability and resilience. Phylum-level community profiles revealed an elevation in dominance of Proteobacteria (ANCOM-BC *p*-value <0.001) and Bacteroidota (ANCOM-BC *p*-value = 0.04) and lower relative abundance of Cyanobacteria (ANCOM-BC *p*-value = 0.01) in the starvation group compared to control and recovery groups. The most abundant genus-level shifts revealed relative increases of the heterotroph *Halioglobus* (*p*-value < 0.05) and lowered abundances of the autotroph *Synechococcus CC9902* in the starvation group. Furthermore, a SparCC correlation network identified co-occurrence of a cluster of genera with elevated relative abundance in the starved mussels that were positively correlated with *Synechococcus CC9902*. The findings from this work provide the first insights into the effect of starvation on the resilience capacity of *Perna canaliculus* gut microbiota, which is of central importance to understanding the effect of food variation and limitation in farmed mussels.

## Introduction

New Zealand green-lipped mussels (*Perna canaliculus*) are commonly found within intertidal and subtidal coastal habitats. This species is also cultivated on marine farms which support a growing aquaculture industry worth over NZ$300 million in export revenues (Aquaculture New Zealand 2020). Given their ecological and economic importance, monitoring the health of wild populations and maintaining the health of cultivated stocks is of utmost importance. However, food availability may vary in quantity and quality within short- (hours) and long-term (seasons) temporal scales, resulting in variable host nutritional states and potential susceptibility to pathogens. Indeed, periods of starvation may play an important role in determining ‘tipping points’ for mussel populations to survive or succumb to other factors thought to be involved in incidences of mass mortalities (e.g. disease, marine heatwaves). Previous research has shown that under starvation stress, insufficient energy intake may negatively affect protein synthesis in mussels, leading to thinner byssal threads and reduced breaking force—these directly increase the risk of mussels detaching from the attachment substrate (Zheng et al. 2022). One to 2 weeks starvation of *Perna canaliculus* also results in a 15–40% reduction of their carbohydrate stores, which is thought to make them more susceptible to stressors (e.g. transport stress and air exposure) (Supono et al. 2022). Mussels filter large volumes of seawater, capturing different types of particulate matter and microorganisms (Neori et al. 2004; Pagano et al. 2016). Therefore, these filter-feeding bivalve molluscs are in constant and direct contact with a dynamically shifting aquatic microbial environment (Glasl et al. 2016; Pita et al. 2018). Host-microbe interactions are thought to play a key role in maintaining mussel health and organ-level functioning, but exposure to pathogenic microbes in the environment may lead to deleterious outcomes. Currently, only one study has profiled the microbiota associated with multiple-tissues of the *Perna canaliculus* (Li et al. 2022), but little is known about the composition of the mussel’s microbiome under various health states.

Some aquatic animals (e.g. the Yellowtail amberjack [*Seriola Ialandi*]) can adapt to sudden short-term dietary changes or starvation via metabolic and physiological adjustments, as well as community shifts in their gut microbiomes (Furet et al. 2010; Xia et al. 2014). For example, fish can survive with limited food resources by utilising alternative energy sources, such as ketone bodies, fatty acids and nitrogenous compounds produced by microorganisms in their gut (Kohl et al. 2014; Kohl and Carey 2016; Barreto-Curiel et al. 2017; Egerton et al. 2018). However, prolonged absence of food and nutrients may lead to shifts in microbial diversity and composition in the gut (Kohl et al. 2014). Alterations in structure and function of the microbial community affects the intestinal immune system and can stimulate inflammatory responses in aquatic animals (Bailey 2014). In turn, a weakened immune system may lead to increased susceptibility to pathogen infections and other stresses, such as temperature, salinity and pollutants, which compounded, may result in lower mortality thresholds (Dehler et al. 2017; Karl et al. 2018; Kers et al. 2018; Zha et al. 2018; Mir et al. 2019). Indeed, previous studies have shown that food limitations in juvenile *Perna canaliculus* mussels reduce their ability to cope with heat stress (Delorme et al. 2020). Additionally, starvation results in lowered oxygen consumption in blue mussels (*Mytilus edulis*) leading to significantly reduced behavioural activity (Tang and Riisgård 2018). However, little is known about the long-term impacts of starvation on the microbiome and health of mussels in general. Thus, the aims of this study were to (1) profile the gut microbiome of healthy mussels under normal feeding, starvation and post-starvation recovery states; (2) identify the microbiome differences among these mussel groups; and (3) identify key patterns in microbiome alterations indicative of the effect of prolonged starvation and post-starvation recovery.

## Material and methods

### Sample collection

**Fig. 1:**
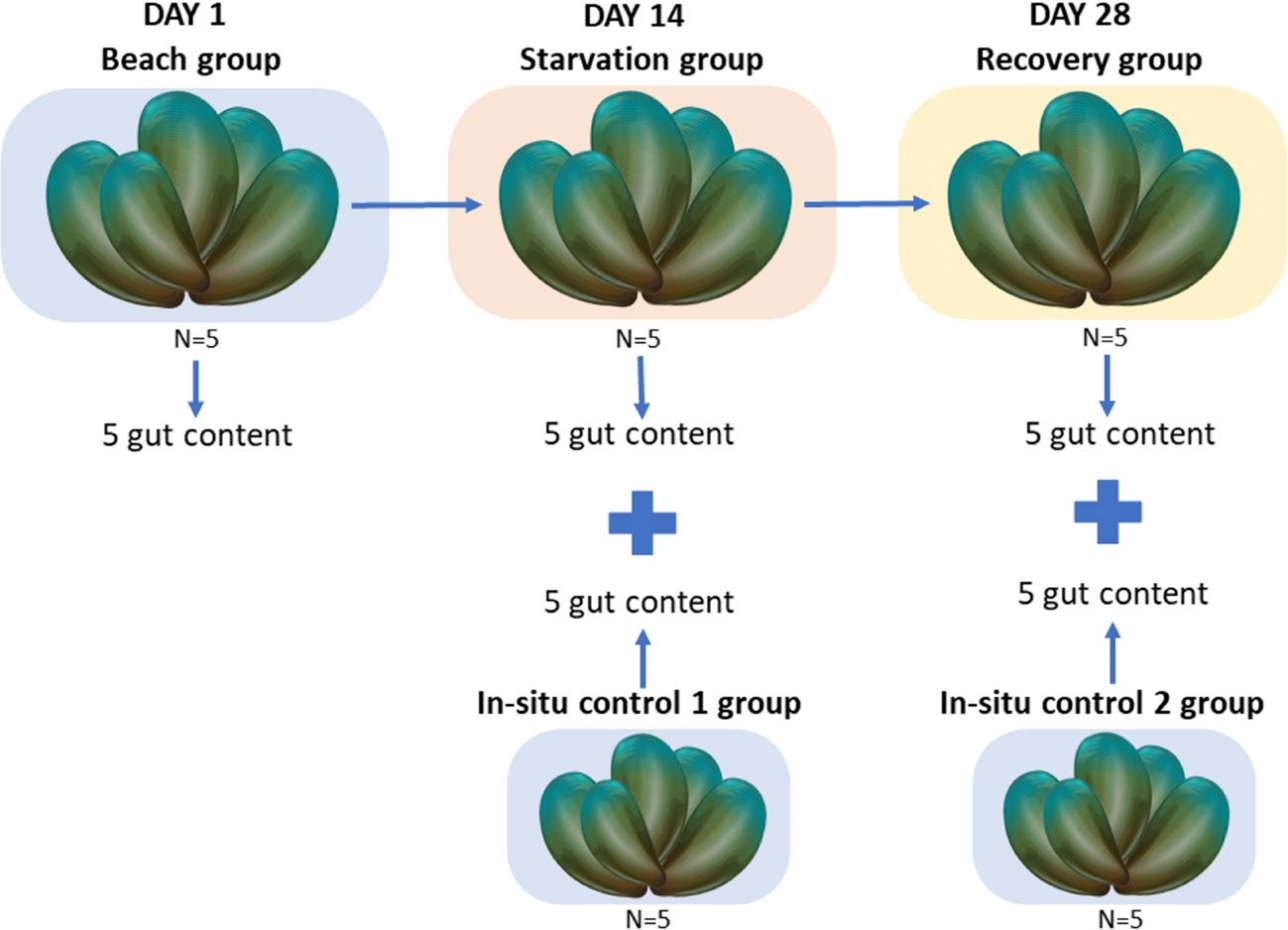
Schematic figure of experimental design: 100 mussels were collected from a natural coastal beach (beach group). Mussels were then subjected to a 14-day starvation in a laboratory controlled static tank system. After 14 days, 5 mussel gut were sampled from the 100 starved mussels (starvation group) alongside 5 more mussels sampled from the original natural location to serve as controls (in situ control 1 group). The rest of the starved mussels were placed back into the original natural beach in a mesh bag and were left to recover. After 14 days of recovery, 5 more mussels were sampled from the mesh bag (recovery group) alongside 5 mussels sampled outside the mesh bag to serve as another control (in situ control 2 group)

Approximately 100 healthy adult mussels (length = 92.8 mm ± 5.9; weight = 63.1 g ± 8.7) were collected from a rocky shore near Kaiaua, Firth of Thames, New Zealand (GPS coordinate: −37.0610, 175.3002) in September 2020 (autumn) (Fig. 1). There were no significant environmental changes (i.e. storms, substantial rainfall, high/low temperatures) during the experimental period. Only mussels that were fully submerged during the lowest tide point (subtidal) were collected and immediately transported while submerged in a container with seawater to the marine laboratory at the Auckland University of Technology (AUT), Auckland, New Zealand (approximately 1-h travel time). The mussels were then placed in a static 50-L tank containing 0.5 micron filtered seawater to remove any potential food particles. The water temperature in the tank was maintained at 14°C, and oxygenated with air stones connected to an air pump and water quality was monitored with a YSI 5200A Multiparameter Monitoring and Control Instrument (pH = 8.2, Salinity = 35 ppt, DO = 9mg/L).

In order to collect gut microbiome samples, mussels were dissected by cutting the posterior and anterior adductor muscles using a sterile dissection knife. The mantle was peeled open with sterile forceps revealing the inner cavity. A cut was then made anteriorly near the oesophagus followed by another cut made posteriorly near the gastro-intestinal tract segment to isolate the main stomach and the digestive gland. Then, the gut content (20–30 mg) was carefully removed from the mussel using sterile disposable forceps and transferred to sterile 2-mL cryovials (BioStor™) containing 20 μL RNA stabiliser (Qiagen, Germany), and immediately snap-frozen in liquid nitrogen and stored at −80°C until further analyses.

### Experimental design

Five mussels were randomly selected (beach group) and dissected to collect microbiome samples from the gut contents. The rest of the mussels were left in the tank without any food (food-deprived animals). The water in the tank was changed every 2 days to maintain good water quality parameters. After 14 days of starvation, five additional mussels were randomly selected (starvation group) for microbiome sampling in the same manner as the initial beach group. On the same day (day 14), another five mussels were collected from the same rocky shore location near Kaiaua, Firth of Thames, New Zealand, transported to the marine laboratory at AUT and sampled (as above) to serve as an in situ beach control group (in situ control 1 group). The in situ control group were set up to control for any potential changes (e.g. time factor or weather) in the environment. Additionally, on day 14, the leftover starved mussels in the tank were then transported from the laboratory back to the rocky shore where they had been collected from, and placed in a mesh bag (2-cm-wide openings) that was submerged and secured to the benthos. These mussels were allowed to recover from the starvation period. After 14 days of recovery time in the wild, five mussels randomly selected from the mesh bag (recovery group) and another five mussels from the surrounding area where the mesh bag was (in situ control 2 group) were collected and transported back to the marine laboratory and sampled for gut microbiome analyses.

### Microbial DNA extraction, PCR amplicon and sequencing

Frozen samples of gut contents were thawed and then homogenised using a FastPrep 24 system (MP Biomedicals; Irvine, California) at six movements per second for 1 min prior to sub-sampling of uniform 250 μL volumes for DNA extraction. The total microbial DNA was extracted from the gut content samples using the DNeasy PowerSoil kit (Qiagen, Germany) following the manufacturer’s instructions with the elution step repeated twice with 50 μL Tris elution buffer. Extracted DNA samples were stored at −20°C before subsequent processing.

Purified DNA samples were quantified using a Qubit 2.0 Fluorometer (Invitrogen; USA). MiSeq (Illumina, USA) libraries were prepared as per manufacturer’s protocol (16S Metagenomic Sequencing Library Preparation; Part # 15044223; Rev. B [Illumina; San Diego, CA, USA]) and as previously described (Archer et al. 2020). PCR analyses were conducted with primer sets targeting the V3-V4 regions of the bacterial 16S rRNA gene: PCR1 forward (5′ CCTACGGGNGGCWGCAG 3′) and PCR1 reverse (5′ GACTACHVGGGTATCTAATCC 3′).

### Bioinformatics and statistical analysis

The total bacterial sequence library size was 12,027,554 before filtering, and 4,727,038 read pair sequences passed quality filtering. Data were pre-processed using our established workflow (Archer et al. 2020). Briefly, 16S rRNA gene were processed using the R package DADA2 v1.8 (Callahan et al. 2016) and cutadapt v3.4 (Martin 2011) to remove forward (CCTACGGGNGGCWGCAG) and reverse (GACTACHVGGGTATCTAATCC) primer sequences for 16S rRNA genes. Bacterial reads were uniformly trimmed to 280 bp (forward) and 250 bp (reverse) and then filtered by removing reads exceeding maximum expected errors of 2 for forward reads and 5 for reverse reads or reads containing ambiguity N. High-quality bacterial reads were then clustered into amplicon sequence variants (ASVs). The resulting taxa were subsequently process using R v3.5.2 (R Core Team 2020). A total of 1386 amplicon sequence variants (ASVs) were inferred from high-quality bacterial reads, which were assigned taxonomic ranks using R package DADA2 v1.8 (Callahan et al. 2016) and SILVA nr v132 database (Quast et al. 2013). MicrobiomeAnalyst (Dhariwal et al. 2017) was used to calculate alpha diversity and beta diversity; differences in group means/median were tested via Kruskal-Wallis. Multivariate interrogation of bacterial profiles were conducted using principal coordinates analysis (Bray-Curtis dissimilarity; tested using permutational MANOVA), hierarchical cluster analysis of samples (Bray-Curtis dissimilarity; Ward linkage), heatmap with combined hierarchical cluster analysis of bacterial genera (Euclidean distance; Ward linkage), and co-occurrence network analysis based on sparse correlations for compositional (SparCC) data and using 100 permutations (correlation inclusion criteria: absolute *r*-value > 0.6, *p*-value < 0.05). The R packages phyloseq (McMurdie and Holmes 2013) and ggplot2 (Wickham 2011) were used to compare and visualise relative bacterial abundances differentials, and the R package ANCOM-BC (Lin and Peddada 2020) was used to test statistical significance of differential taxon abundances between sample groups.

## Results

### Richness and diversity of microbial communities

**Fig. 2:**
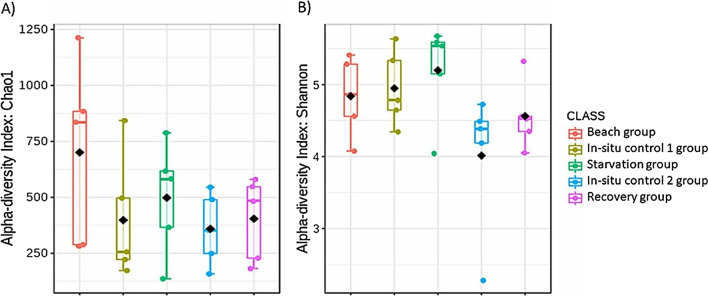
Richness and diversity indices for **A** Chao1 and **B** Shannon at ASV level. Black diamonds represent the mean of the data

**Fig. 3:**
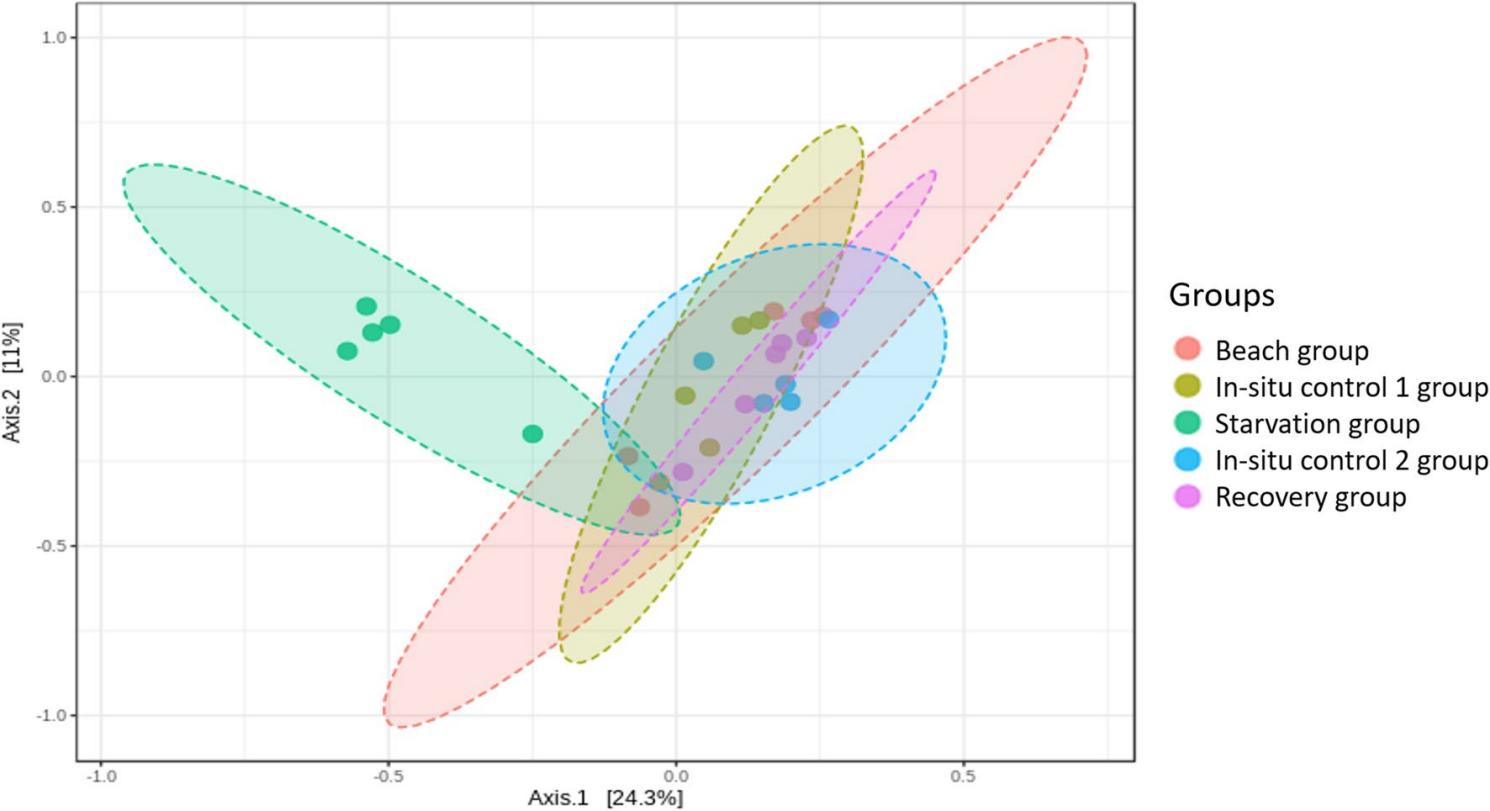
Principal coordinates analysis (PCoA) of mussels in the different groups based on gut bacterial profiles (ASV). Eclipses represent 95% confidence

Amplicon sequencing of gut contents from all mussel samples generated a total sequence library size of 12,027,554 with 4,727,038 paired-end sequences passing quality filtering. High-quality reads were clustered into 1386 bacterial ASVs. There was no significant difference in microbial richness or diversity between the sample groups for Chao1 (Kruskal-Wallis *p*-value: 0.40567) and Shannon (Kruskal-Wallis *p*-value: 0.15804) indices, respectively (Fig. 2). However, a principal coordinate ordination analysis (PCoA) at the ASV level highlighted that the bacterial profiles of the starvation group were distinctively clustered apart from the other sample groups (*F*-value: 2.567; R-squared: 0.34; *p*-value < 0.001) (Fig. 3). The permutational MANOVA yielded an R-squared value of 0.46 and *p*-value < 0.001.

### Microbial community structures analysis

**Fig. 4:**
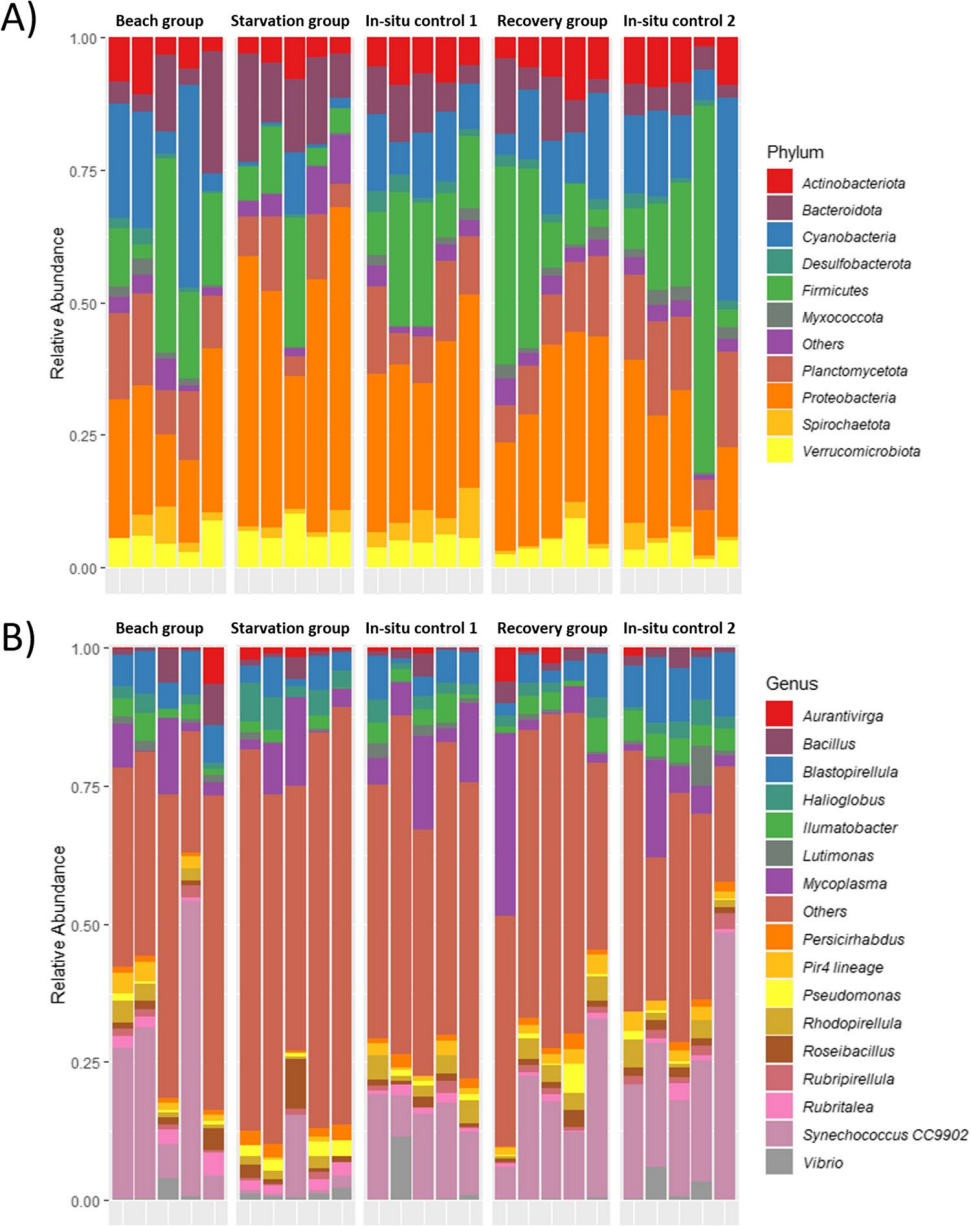
Phylogenetic classification of the bacterial communities: **A** at the phylum level, and **B** at the genus level

Phylum-level community profiles at the different experimental points revealed distinct bacterial compositional patterns, particularly separating the starvation group from the others (Fig. 4a). Most noticeably, an elevation in dominance of Proteobacteria (beach group = 24%, starvation group = 47%, recovery group = 25%; ANCOM-BC *p*-value < 0.001) and Bacteroidota (Beach group = 5%, starvation group = 12%, recovery group = 6%; ANCOM-BC *p*-value = 0.04) and lower relative abundance of Cyanobacteria (beach group = 24%, starvation group = 4%, recovery group = 19%; ANCOM-BC *p*-value = 0.01) were observed in the starvation group compared with all other sample groups. Further analysis of the most abundant genera across the groups revealed that in the Starvation group, *Halioglobus* were richer (*p*-value = 0.001) and had higher abundance compared to the other groups, whereas the relative abundance of *Synechococcus* CC9902, a photosynthetic marine plankton (Kim et al. 2018), was lower (*p*-value = 0.04) in the starvation group (Fig. 4b).

**Fig. 5:**
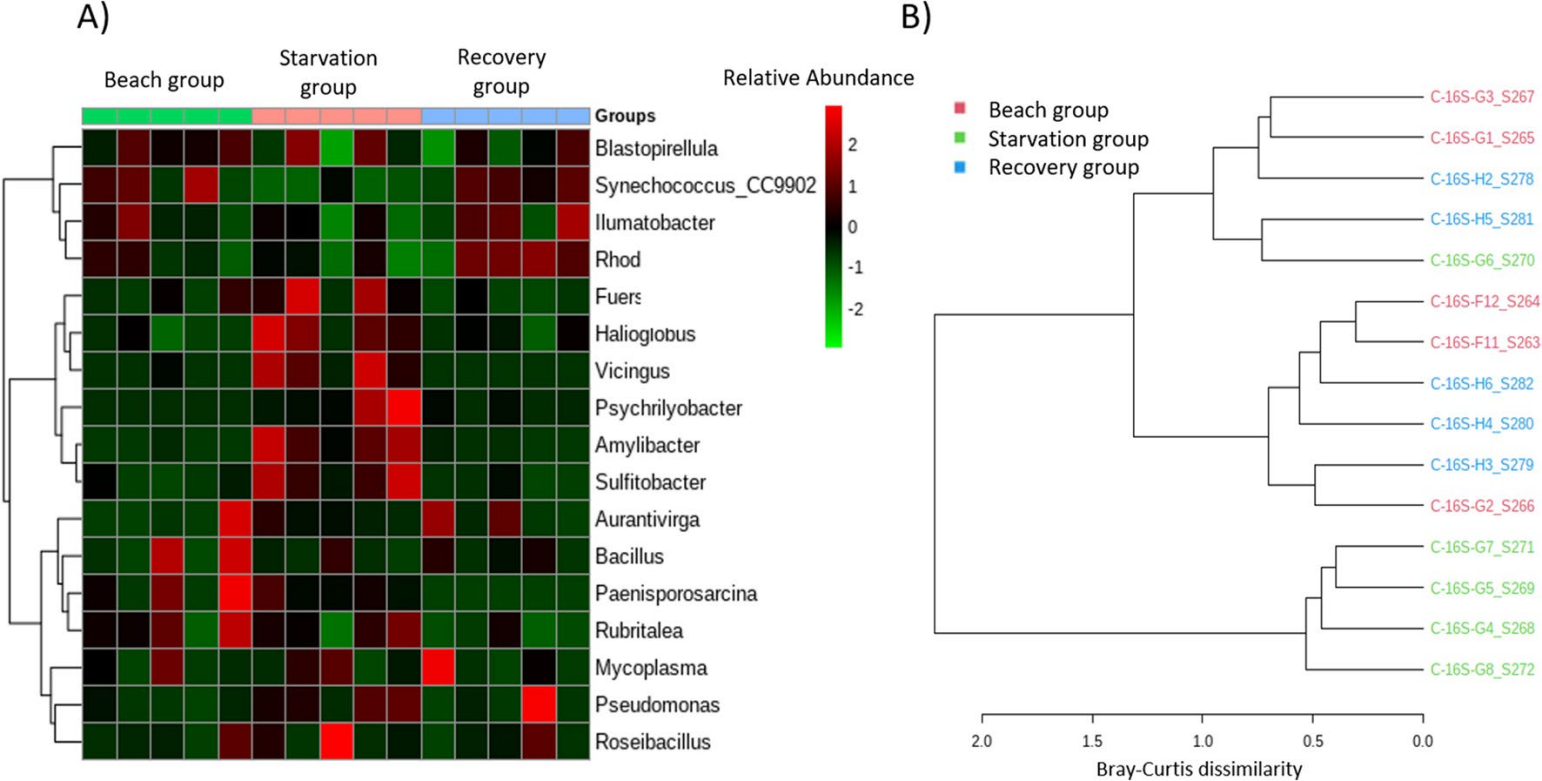
**A** Heatmap and cluster analysis of the top 20 (3 unclassified genera were removed) most abundant genera. Bacterial genera are shown row-wise, samples are shown column-wise and coloured by scaled relative abundance. **B** Hierarchical clustering dendrogram (Ward algorithm) constructed via Bray-Curtis distances of mussel samples based on genera

**Fig. 6:**
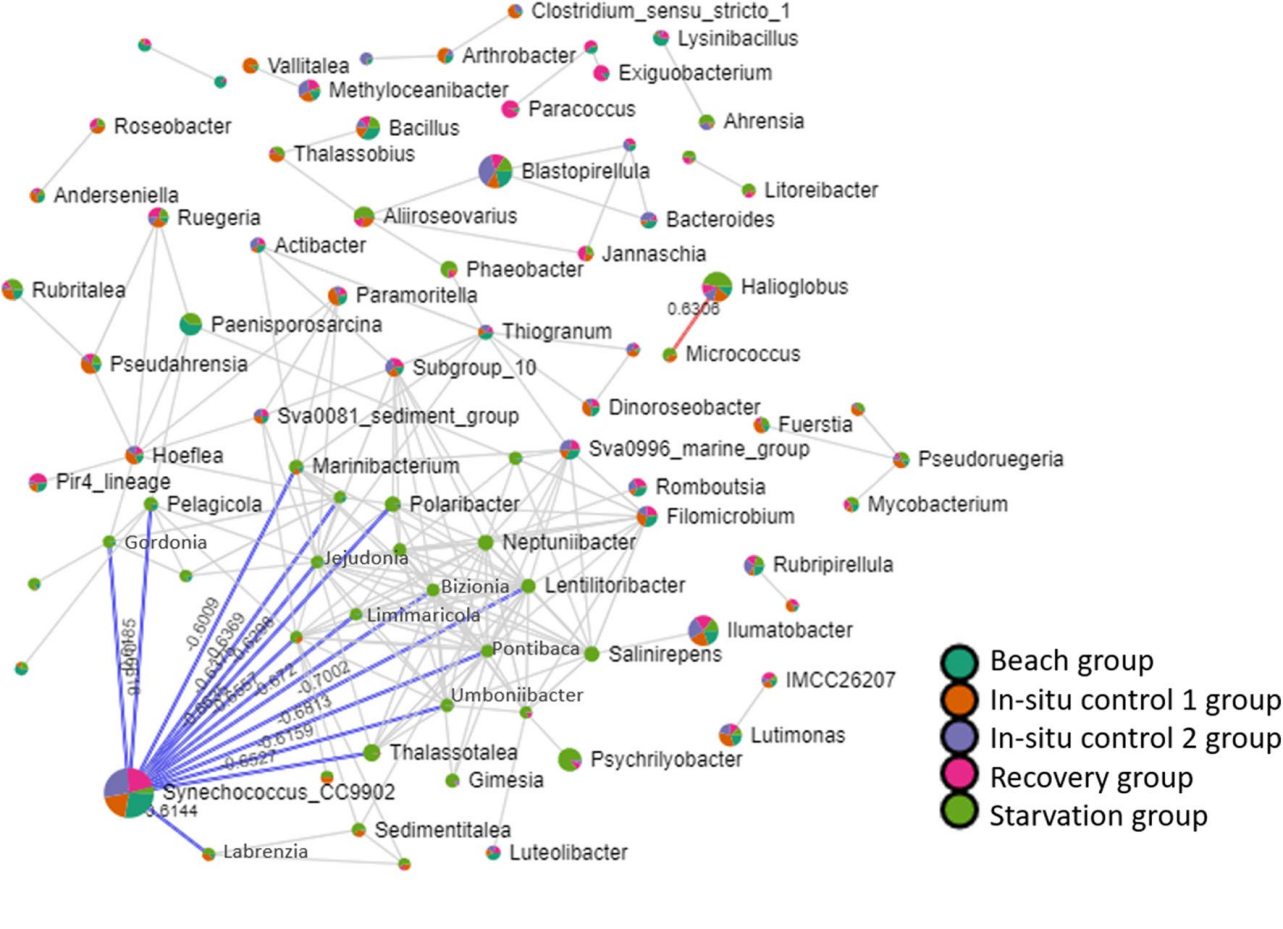
Co-occurrence network analysis of significant correlations (SparCC; absolute *r*-values > 0.6; *p*-value < 0.05) between genera. Each node represents a bacterial genus and the edges represent the correlation coefficients between the genera. Synechococcus (CC9902) were selected as a significant hub genus and the blue edges from this node represents negative correlations between Synechococcus_CC9902 and other genera. The values on the edges signify the correlation coefficients. Nodes are coloured according to the groups (beach group, in situ control 1 group, in-situ control 2 group, recovery group and starvation group)

Clustering of the top 20 most abundant genera revealed four bacterial genera which belonged to Proteobacteria (Fig. 5a). These genera, including *Halioglobus*, *Amylibacter*, *Sulfitobacter* and *Vicingus* were clustered together and had higher relative abundances in the Starvation group. Finally, the bacterial profile dissimilarity at the genus level between the starved mussels and those in other groups were further highlighted via a dendrogram (Fig. 5b), with most members of the starvation group being clustered separately. Additionally, the SparCC network analysis returned a complex structure depicting the interactive associations of the prevalent genera with others (Fig. 6). Interestingly, we observed that *Synechococcus* CC9902 had significant negative correlations with 14 genera (*r*-values > 0.6 and *p*-values < 0.01). The colour of the nodes in the lattice indicated the occurrence of these specific 14 genera are elevated in starved mussel, forming a co-occurrence cluster.

## Discussion

Shellfish such as mussels can experience long periods of food limitation which may lead to starvation. However, knowledge on how the mussel gut microbiota responds to starvation remains scarce. This study provides the first exploratory analysis into the effect of starvation on the gut microbiome of *P. canaliculus*. The microbiome profile of mussels prior and post-starvation was compared and analysed to identify microbiome structural changes, key taxon and taxon interactions, and host-microbiome interactions indicative of stress levels and scope for recovery.

Richness and diversity of gut microbial communities were similar among groups of mussels before, during and after starvation. This is interesting since an artificial marine environment was created in the laboratory to starve the mussels, and previous studies have shown that artificial environments rarely maintain the same bacterial diversity as the original or natural environments (Patin et al. 2018). However, our results revealed no statistically significant shifts in alpha diversity between the starvation and control groups. The stability of bacterial richness may not be a good indicator for the effect of starvation on bacterial counts as opposed to other effects, such as seasonal (Häder et al. 1998) and temperature (Tiefenthaler et al. 2009; Vargas et al. 2021) changes. A potential reason for this result could be the re-stabilisation capacity of *P. canaliculus* microbiome towards the effects of starvation. Indeed, a study on zebrafish have found that long-term starvation of 21 days induces progressive changes in microbiome composition and host gene expression in the zebrafish intestine, and these changes are rapidly reversed after refeeding (Jawahar et al. 2022). Some aquatic animals can utilise nitrogenous compounds produced by various microorganisms in their gut as an alternative energy source during short-term starvation (Kohl et al. 2014; Kohl and Carey 2016; Barreto-Curiel et al. 2017; Egerton et al. 2018), hence promoting the diversity of such microorganisms. However, mussels which were starved had a distinct microbiome profile with a short-term effect on gut microbiome composition that was indicative of the starvation state*.* The mussel microbiome reverted to that of pre-starvation state after only 14 days, matching the microbiome profile of the in situ controls. These findings suggest a fast recovery and further highlights the potential inherent resilience of the species to intermittent food supplies or starvation stresses during periods of low food availability.

The microbiome communities largely altered in response to host starvation were relative abundances of Proteobacteria (gamma), Bacteroidetes, Firmicutes and Cyanobacteria phyla. The most indicative change in phyla associated with the starvation period was an increase in the abundance of Proteobacteria (gamma). These shifts are consistent with studies on the effect of starvation of zebrafish (*Danio rerio*) (Semova et al. 2012) and grass carp (*Ctenopharyngodon idellus*) (Tran et al. 2018). Previous studies have also reported that Proteobacteria were associated with unstable gut microbiota, energy instability and inflammation (Shin et al. 2015; Tran et al. 2018). The increased Proteobacteria in post-starvation mussels followed by its decreased abundance in post-recovery mussels suggests the association of Proteobacteria with starvation state, and the mussel’s ability to rapidly restore its intestinal microbiota and energy cycle. The Phylum Bacteroides was also found to have higher relative abundance in starved *P. canaliculus*. However, there are contradicting reports on whether this group of bacteria is commonly associated with starvation, and may highlight Bacteroides as a poor signature of the starvation condition. For example, Nile tilapia (*Oreochromis niloticus*) were shown to have lower abundance of Bacteroidetes after 14 days of starvation (Sakyi et al. 2020), whereas starvation in loach (*Paramisgurnus dabryanus*) revealed a higher abundance of Bacteroidetes (Peter et al. 2020). A large part of the proteins made by the Bacteroides genome are able to break down polysaccharides and metabolise their sugars (Xu et al. 2003). They play a fundamental role in the processing of complex molecules to simpler ones in the host intestine. Their ability to harvest alternative energy sources from food might allow Bacteroides to be more competitive than other bacteria in the *P. canaliculus* gut during starvation (Flint et al. 2012; Lapébie et al. 2019). Finally, a significantly lower relative abundance of Cyanobacteria was detected in the starved mussels. This result was not surprising considering that the Phylum Cyanobacteria is a dominant group in the water column where they form an important part of the phytoplankton (Moore et al. 2019). Cyanobacteria are rich in proteins and contain carotenoids, vitamins, minerals and essential fatty acids (Wells et al. 2017), and contribute significantly to the diet of most bivalve filter feeders. Some bivalve species, such as the swan mussel (*Anodonta cygnea*), have been shown to preferentially select Cyanobacteria from the water column to boost their nutritional state and contribute to the accumulation of energy reserves during host gametogenesis (Lopes-Lima et al. 2014). Although there were no mentions of the mussels being under stress by the author, the study has shown that Cyanobacteria is an important food source for the mussels. Therefore, a decrease in Cyanobacteria in starved *P. canaliculus* can potentially be reflective of the mussel’s energy deprivation and subsequently hinder its reproductive function. Another area for potential future work is to perform functional analysis on these community changes as only specific taxa within these groups have known functions.

At the genus level, the most obvious microbiome community alterations can be seen in the increase in *Halioglobus* (ANCOM-BC *p*-value=0.01) and decrease in *Synechococus* (strain CC9902) (ANCOM-BC *p*-value=0.01) in the starvation group. These species are involved in various chemical processes and have mostly been recorded in ocean and coastal areas. For example, *Halioglobus* was previously isolated from the coast of Japan and has been shown to have 18 genes related to denitrification (Park et al. 2012). *Synechococcus* cyanobacteria are one of the most important components of photosynthetic picoplankton (Partensky et al. 1999; Flombaum et al. 2013; Sohm et al. 2016), and their lowered abundance is reflective of this food source being unavailable to the mussels in the starvation group. However, information on host-microbiome interactions of these genera and how they are modulated by host starvation remains scarce in molluscs. Therefore, in this study, we performed exploratory investigations on bacterial co-occurrence via SparCC at the genus level to provide novel information on the underlying correlations of microbial community structure with regard to mussel starvation and post-starvation recovery. Bacteria interact extensively within and among species while responding to external stimuli. They can perceive neighbouring cells and the environment and often reflects this within the content of bacterial genomes (Bassler and Losick 2006). Furthermore, the dynamics of bacterial communities are determined by pairwise interactions that occur between species in the community (Stubbendieck et al. 2016). In the present study, our results further highlight the genus *Synechococus* (strain CC9902), and its association to multiple genera during mussel starvation. Indeed, this subset of genera has highly elevated community abundance during starvation. Although functional insights of these genera are currently lacking, our network association analysis offers a starting point to seek metabolic implications for these correlations and their roles on host physiology.

## Conclusions

We identified a microbial community dominated by Proteobacteria, Bacteroidota and Cynaobacteria that rapidly shifted during mussel starvation then quickly returned to their natural state as mussels recovered from starvation. Specific shifts in bacterial communities appear to be a response to starvation, potentially associated with the host’s energy stability and absorption from lack of food source. Our results highlight lowered community composition of Synechococus (strain CC9902) and its co-occurrence with multiple genera which were elevated during mussel starvation. These results offer insights into the effect of starvation on subsets of genus-level compositions and associations with one another. The findings of this study provide new knowledge on host–bacteria interactions within the gut microbiome in *P. canaliculus* during starvation stress and recovery. This study also provides a foundation for potential future research such as functional analysis on the above mentioned community changes as well as the opprtunitity to examine metabolic implications from correlations identified in this study.

## Data Availability

Raw data and output are available on reasonable request to the corresponding author.
